# The promise of competency-based education in the health professions for improving global health

**DOI:** 10.1186/1478-4491-10-43

**Published:** 2012-11-16

**Authors:** Larry D Gruppen, Rajesh S Mangrulkar, Joseph C Kolars

**Affiliations:** 1 Department of Medical Education, University of Michigan Medical School, G1113 Towsley Center, 1500 E. Medical Center Drive, Ann Arbor, MI, 48109-5201, USA; 2 Department of Internal Medicine, University of Michigan Medical School, 3110 Taubman Center, SPC 5368, 1500 E. Medical Center Drive, Ann Arbor, MI, 48109-5201, USA

## Abstract

Competency-based education (CBE) provides a useful alternative to time-based models for preparing health professionals and constructing educational programs. We describe the concept of ‘competence’ and ‘competencies’ as well as the critical curricular implications that derive from a focus on ‘competence’ rather than ‘time’. These implications include: defining educational outcomes, developing individualized learning pathways, setting standards, and the centrality of valid assessment so as to reflect stakeholder priorities. We also highlight four challenges to implementing CBE: identifying the health needs of the community, defining competencies, developing self-regulated and flexible learning options, and assessing learners for competence. While CBE has been a prominent focus of educational reform in resource-rich countries, we believe it has even more potential to align educational programs with health system priorities in more resource-limited settings. Because CBE begins with a careful consideration of the competencies desired in the health professional workforce to address health care priorities, it provides a vehicle for integrating the health needs of the country with the values of the profession.

## 

Improvements in global health can only be realized through the development of a workforce that has been educated to promote health and to care for those with disease [1]. Increased attention is being placed on competency-based education as a means for optimizing the preparation of health professionals. Competency-based education (CBE) is a framework for designing and implementing education that focuses on the desired performance characteristics of health care professionals. Although ‘competence’ has always been the implicit goal of more traditional educational frameworks, CBE makes this explicit by establishing observable and measurable performance metrics that learners must attain to be deemed competent. By contrast, more traditional frameworks have delineated the intended learning objectives of instruction [2-7] Learning objectives often focus on what the learner should ‘know’ whereas competencies focuses on what the learner should be able ‘to do’, (while acknowledging that learning objectives are often requisite but typically in and of themselves insufficient).

There have been many definitions of ‘competence’ and ‘competencies’, all sharing many common features [6-9]. For this paper, we use Albanese *et al*.’s [10] five characteristics to define a competency:

## A competency focuses on the performance of the end product or goal state of instruction

**Figure 1:**
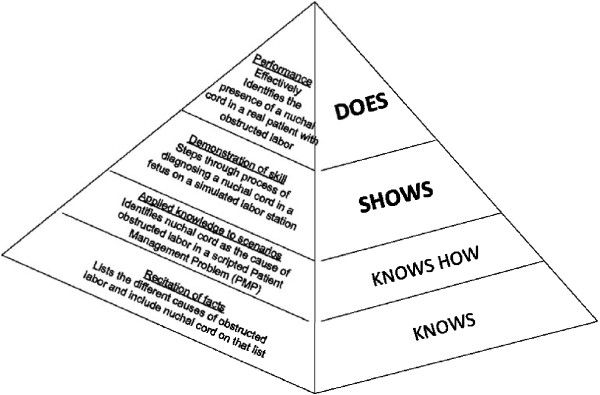
**Miller**’**s pyramid **[11].

Traditional education tends to focus on what and how learners are taught and less so on whether or not they can use their learning to solve problems, perform procedures, communicate effectively, or make good clinical decisions. By emphasizing the results of education rather than its processes, CBE provides a significant shift in what educators and policy makers look for in judging the effectiveness of educational programs. Figure 1 illustrates Miller’s pyramid [11], which describes the differing levels of educational goal states. For early learners, outcomes at the level of ‘knows’ and ‘knows how’ may be sufficient, but for more advanced learners, educational goals are more typically at the levels of ‘shows’ and ‘does’. These higher levels of the pyramid reflect performance in practice, not just in the classroom. In CBE, the critical issue is that the learner reaches the specified level of performance in a competency; how he or she reaches that point (the educational process) is secondary. 

## A competency reflects expectations that are external to the immediate instructional program

Traditional educational programs too often have an insular character in which the expectations of learners are based on what has been taught in the past. In CBE, success is determined by the ability to perform to expectations that are largely determined by stakeholders outside of the educational program itself.

## A competency is expressible in terms of measurable behavior

Although traditional education does assess learner knowledge and progress, CBE places a much higher premium on learner performance of tasks and activities representative of the competencies. These assessments emphasize behavioral measures that depend on integrating knowledge and skills derived from an aggregate of educational experiences and parts of the curriculum [4].

## A competency uses a standard for judging competence that is not dependent upon the performance of other learners

Each performance assessment of competence must be accompanied by an explicit criterion for determining whether or not a given learner has or has not attained the required level of performance to be considered ‘competent’. These criteria or performance standards are not determined by the performance of other learners (that is, not graded on a ‘curve’) but by the expert judgment of practitioners and educators in the field. Thus, it is desirable that ALL learners will achieve ‘competence’ after training.

## A competency informs learners, as well as other stakeholders, about what is expected of them

By focusing on the outcomes of education, CBE is often much more transparent and therefore accountable to learners, policy makers and other stakeholders. Indeed, defining a discipline’s values, goals and priorities is an implicit part of defining competencies, which enables the competencies to communicate these values and expectations to various stakeholders within and outside the discipline.

### Defining the curriculum for competency-based education

**Figure 2:**
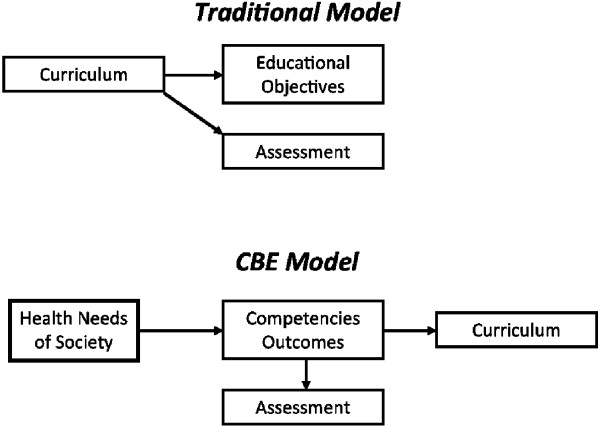
**Comparing traditional and competency**-**based educational models^a^**.

The curriculum, or what is to be learned, is at the heart of all educational models. It is the genesis or origin of the curriculum that differentiates traditional models from CBE. Traditional curricula often become anchored to historical legacies that codify the traditions, priorities, and values of the faculty in that profession. Learning objectives are often defined to reflect what the faculty desire to teach or deem important rather than the other way around. This ‘curriculum driven’ definition of learning outcomes often fails to coincide with the needs of society. For example, curricula designed in resource-rich settings may be projected or perceived as ‘gold standards’ for resource-poor settings, to the exclusion of other necessary topics that are more likely to address local health needs. Competency-based education places the curriculum as an ‘end product’ of a needs assessment rather than as the structure that constrains educational objectives and assessments (see Figure 2).

When comparing CBE to a traditional model of education (Figure 2), three fundamentally different characteristics emerge. First, CBE explicitly maps the specific health needs of the populations to a set of competencies for the workforce to be trained. For example, the United States National Board of Medical Examiners, which is responsible for the licensing of all graduating physicians in the United States, has adopted a plan to realign its licensure processes to more substantially reflect the expectations of patients [9]. Second, CBE uses these expectations to then develop and implement learning experiences (the curriculum) designed to produce the requisite knowledge, values, and skills in the learners to achieve these competencies. Finally, CBE uses the same set of competencies to develop critical assessment programs to determine the extent to which they are reached.

### The practical steps in implementing a competency-based educational system

The past several years have seen considerable growth in the development of competencies in different health professional fields, including medicine, nursing, midwifery, and public health. However, many of these efforts fall short of a fully implemented CBE model because of challenges in four domains.

## Defining the health needs of the community

A CBE program has the potential to improve the health of the community it serves only to the extent that it uses context-specific health issues to determine the desired competencies [1]. Explicitly defining the health needs of the community is necessary in order to identify outcome variables that can be mapped to desired changes in health, to ensure program accountability to relevant stakeholders, and to focus learners in these health professions on aligning their own performance with the health expectations of society.

Needs assessments that reflect available health data, input from the community and the public health perspective are necessary to inform this process. A multitude of approaches have been used for this, including a carefully designed needs analysis of key informants [12], direct observation of health care providers in practice along with surveys of practitioners regarding perceived need [13], multilateral expert panels [14], and national consensus-building processes [15].

Still, there is a tendency for educational institutions to focus on their own narrow mission and definition of competence. In order to incorporate the health needs of the larger community into the competency definition, several models to explicitly elicit these needs are possible. One could be establishing a national health competency board with broad stakeholder representation, which would set competencies for the country as a whole. A second might include explicit partnerships with health workers in the field as a means of validating the relevance of competencies and ensuring key domains are not neglected. A third could be a responsive feedback system in which graduates report back to the school the adequacy of their preparation in regard to the competencies and the need for modifications. The specific methods for incorporating community health needs will necessarily depend on the unique characteristics of the country or region, but educators and health care practitioners must be creative in ensuring that this critical step takes place and that it reflects the input of wide spectrum of stakeholders.

## Defining competencies

The central step in shifting from a traditional to a competency-based educational framework is to define the learner competencies. These competencies reflect specific goals of education, but also express institutional, disciplinary, or national priorities. Competency definitions are intended, among other things, to communicate these priorities in memorable and meaningful ways.

Schools, licensing agencies, and professional societies may each define the competencies differently or use different terminology for similar domains or even have different conceptions of what constitutes a ‘competent’ professional. For example, each of five published competency frameworks includes ‘communication skills’. However, only two contain competencies related to ‘managing information’ or ‘lifelong learning’. Other competencies, such as ‘clinical skills’ are represented in each set but under somewhat different labeling.

Competency descriptions typically operate at multiple levels of detail. ‘Communication skills’, as a description of a target competency, does not provide clear guidance for educators or learners. To make competencies relevant to education, they must be translated into much more specific statements that include the context, content, and criteria for the competency to be attained. This results in a hierarchy of competency specificity within any single domain.

For example, the University of New South Wales [16] identifies ‘effective communication’ as one of its eight competencies, but then goes on to refine this to include three more detailed competencies: ‘communicates effectively with patients and their families’, ‘communicates effectively with peers and tutors’, and ‘communicates with communities’. There is then further specificity with more operational competencies such as ‘counsels patients appropriately on a range of health risks including poor nutrition, smoking cessation, drug and alcohol management, and refers to community programs and services if appropriate’. It is easier to develop educational activities and assessment tools for the more specific, detailed competency statements than for the more broad domains.

This example illustrates a major challenge in CBE, which is the rapid expansion of the number of competencies as they become more focused on teachable and observable skills or performance. This creates an information burden for learners and the institution. It may also lead towards ‘checkbox education’ and a focus on individual pieces of performance with the loss of the more holistic, comprehensive competencies we desire in professionals. Checkbox education is not, however, an inherent limitation of CBE. For example, proficiency in patient care, medical knowledge and communication all might readily be assessed through the observation of a learner performing a normal delivery of a newborn. This reflects the proposal of ten Cate and colleagues that we use a more integrative framework for assessing competencies that centers around ‘entrustable professional activities’, those that reflect day-to-day professional activities appropriate to the level of learner [4].

## Self-regulated and flexible learning options

Competency-based education promotes a necessary flexibility in the time and sequence of what is to be learned that is regulated by the needs of the learner [17]. Therefore, CBE allows for a highly individualized learning process rather than the traditional, lock-step, one-size-fits-all curriculum [18]. Ideally, students would have an opportunity to explore a menu of choices in learning activities and methods that could allow them to achieve competency.

By way of example, Table 1 depicts competencies mapped to the global health problem of managing maternal obstructed labor, formulated within the 10 competency domains specified by the Indiana University School of Medicine [19]. For each specific competency, a set of both learning and assessment method options are listed, appropriately mapped to the pedagogical framework. As can be seen in Table 1, some competencies may lend themselves to a greater number of learning options than do others. 

**Table 1:** Linking competencies at the abstract and contextualized levels with assessment and learning methods using obstructed labor as an example

**Competency domain [16]**	**Competency in context: obstructed labor**	**Learning method options**	**Possible assessment method**
Effective Communication	The learner explains different options for accelerating birth to the mother in a calm, clear manner.	Structured practice using simulated patients. Assigned reading on treatment options.	Standardized patient exercise
Basic Clinical Skills	Using physical examination techniques, the learner identifies the presence of a nuchal cord as the etiology behind obstructed labor.	Simulation/Mannequin practice. Physical exam textbook. Supervised clinical experiences.	Structured direct observation and feedback.
			Standardized patient examination.
Using Science to Guide Diagnosis, Management, Therapeutics and Prevention	The learner identifies community-based resources to assist in the prenatal management of women at risk for obstructed labor.	Self-guided search. Assigned reading.	Written examination
Moral reasoning and ethical judgment	The learner explains the most important competing issues that weigh in the decision to perform life- saving maternal interventions that may place the fetus at risk in obstructed labor.	Small group discussion of case scenarios.	Oral examination
		Programmed reading.	
Problem solving	The learner appropriately identifies and refers high- risk cases of obstructed labor that require subspecialty management	Small group discussion with scripted patient management problems. Assigned problem set with feedback.	Chart audit
Professionalism and role recognition	The learner maintains confidentiality in the care of women with obstructed labor.	Lecture.	Supervisor evaluation
		Self-directed review of confidentiality policy.	

A system in which performance against preset expectations determines progress through a learning program challenges the notion of a ‘time-based’ curriculum and may lead to the situation where ‘time and method are the variables and achievement is the constant’ [20]. The relationship of time and practice to skill development has been well established [21]. However, it is also clear that different learners will require different amounts of time to achieve certain standards of performance. The logistics required to scale the implications of students learning at different rates are substantial. Clustering of learners facilitates faculty and student convenience, as well as efficient space allocation and resource expenditures. However, it also ignores the possibility that some learners may require less time to achieve competence than thought, or that some may be able to ‘test out’ of a given set of educational experiences if they have demonstrated the required level of performance at baseline. As such, learning ‘efficiency’ takes on a vastly different meaning when the time on task is individualized, flexible and variable.

Flexibility and individualized learning place considerable burden on information systems to track and document learner progress through a CBE curriculum. It also requires faculty members to attend to student progress and transitions in ways not often required in traditional curricula.

## Assessing learners for competence

Without evidence of the learner’s ability to fulfill a given competency, it is impossible to judge the success of either that individual or the educational program. The diversity of competencies defined for a given set of learners also requires a diverse set of assessment methods. The contextualized competencies in Table 1 illustrate how different competencies need to be assessed in different ways but also how different methods may be appropriate for the same competency.

The need to match more complex assessment methods with more sophisticated competency outcomes is also challenging. While a multiple-choice examination may be a reliable and accurate reflection of knowledge, it is an inappropriate measure of application and performance in real-world settings. Higher order assessments would require direct observation, structured feedback on performance, or skills-based evaluations in simulated or real patients [22,23]. Without assessment, CBE becomes little more than traditional education with a more clearly defined set of goals and objectives.

In addition to assessment methods, CBE requires clearly specified performance criteria or standards on these assessments that enable faculty to judge that the learner has reached the minimal level of performance that qualifies as ‘competent’. It is important to recognize that standards can be set ONLY after the competencies have been defined and assessment methods developed and applied. These standards may require technically complex procedures [24,25] to define the actual score or performance metric by which a learner would be considered.

### Implications for competency-based education in resource-poor settings

Resources for health are finite and often felt to be insufficient, regardless of the setting. Resource-poor countries have a tendency to try to emulate resource-rich countries with regard to their educational standards and desired health care outcomes. As a result, local educational standards are often driven by the desire to fit into frameworks that are in place elsewhere. While many of the domains of competency, such as professionalism and communication, are universal, many of the more specific competencies in resource-rich settings presume the presence of a particular health care system and an education system in which those competencies can be nurtured and fully appreciated. This may not be the case in resource-poor countries. Furthermore, competence in domains such as professionalism and communication, like all competencies, is very sensitive to the context of the individual and their culture. The language or approach that is used to break bad news, elicit sensitive information, or motivate others to take care of themselves will vary widely with personal attributes and the culture.

While competencies are context-specific, there exist common approaches to CBE. First, as noted earlier, the local health issues and priorities of a country should serve as the starting point. Second, there needs to be a discussion of what kinds of competencies are needed to address these health care priorities. While these will often reflect the major domains described earlier in this article, competencies will need to be very context-specific and take into account the availability of faculty and local resources. Third, it should be clear which health professionals are expected to be able to achieve which competencies. Who should be competent at caesarean section? All graduating physicians? Nurse midwives? Labor technicians? One of the consequences of CBE is that skills that were once considered the domain of only select professions could potentially be ‘task shifted’ to other professions if they are able to perform at the same level of competency. Finally, while the definition of competencies is necessary, it is insufficient unless the metrics of achievement are clearly defined – by what standard do we deem someone competent to be able to do a caesarean section? These competencies should then in turn drive the curricula and the modes of learning.

Resource-poor settings need health professionals who are not just clinically ‘competent’ but who can provide leadership to set expectations and transform health within their country. These skills are particularly needed in resource-poor settings where health care systems have yet to be optimally shaped. A competency-based focus on leadership, policy formation, management, and the direction of interdisciplinary teams is essential for the development of professionals in low-resource settings.

Both resource-rich and -poor countries must recognize the need for faculty development in regard to planning and implementing CBE. CBE is challenging even for schools with adequate faculty numbers, but becomes even more so when faculty resources are inadequate. Because CBE is so different from traditional educational models, few faculty members readily adapt to CBE. This is an area in which shared development and expertise would be fruitful. Collaborations between countries will also aid resource-rich countries to examine their assumptions and more explicitly address the context in which they are defining their competencies, learning from the efforts of colleagues in resource-poor countries.

## Conclusion

Education systems can improve the efficiency and effectiveness of their mission through competency-based education and a focus on the performance requirements for all health professionals. The challenges in implementing such a system are significant, but can be addressed by drawing on existing innovative initiatives, such as work-based assessment methods, developing cadre of faculty with specialized evaluation expertise, and identifying developmental milestones for competencies. Such an approach can move 21^st^ century health professions education into a sphere of increased accountability to society for improving health. Our analysis of the fundamental principles and implications of CBE suggest that it is a viable model for conceptualizing health professions education in resource-poor nations. It has particular appeal as a means of avoiding the possible application of Western educational models to countries and contexts for which those are inappropriate. Because CBE begins with a careful consideration of the competencies desired in the health professional workforce, it provides a vehicle for integrating the health needs of the country with the priorities of the profession. The logistical challenges of implementing CBE are far from trivial, but the model holds potential for more efficient and effective training of health care workers.

## Endnotes

^a^Figure from Gruppen LD, Mangrulkar RS, Kolars JC. Competency-based education in the health professions: implications for improving global health. A report to the Commission on Education of Health Professionals for the 21st Century. June 2010. Figure reproduced in [1].

## Competing interests

The author(s) declare that they have no competing interests.

## Authors' contributions

LDG, RSM, and JCK collaborated equally in the discussions, literature review, conceptualization, and the drafting of the manuscript. All authors read and approved the final manuscript.
